# Nucleation of α-Synuclein Amyloid Fibrils Induced by Cross-Interaction with β-Hairpin Peptides Derived from Immunoglobulin Light Chains

**DOI:** 10.3390/ijms242216132

**Published:** 2023-11-09

**Authors:** Laetitia F. Heid, Tatsiana Kupreichyk, Marie P. Schützmann, Walfried Schneider, Matthias Stoldt, Wolfgang Hoyer

**Affiliations:** 1 Institut für Physikalische Biologie, Heinrich Heine University Düsseldorf, 40204 Düsseldorf, Germany; 2 Institute of Biological Information Processing (IBI-7) and JuStruct, Jülich Center for Structural Biology, Forschungszentrum Jülich, 52425 Jülich, Germany

**Keywords:** β-hairpin, cross-seeding, synucleinopathy, AL amyloidosis, primary nucleation, secondary nucleation

## Abstract

Heterologous interactions between different amyloid-forming proteins, also called cross-interactions, may have a critical impact on disease-related amyloid formation. β-hairpin conformers of amyloid-forming proteins have been shown to affect homologous interactions in the amyloid self-assembly process. Here, we applied two β-hairpin-forming peptides derived from immunoglobulin light chains as models to test how heterologous β-hairpins modulate the fibril formation of Parkinson’s disease-associated protein α-synuclein (αSyn). The peptides SMAhp and LENhp comprise β-strands C and C′ of the κ4 antibodies SMA and LEN, which are associated with light chain amyloidosis and multiple myeloma, respectively. SMAhp and LENhp bind with high affinity to the β-hairpin-binding protein β-wrapin AS10 according to isothermal titration calorimetry and NMR spectroscopy. The addition of SMAhp and LENhp affects the kinetics of αSyn aggregation monitored by Thioflavin T (ThT) fluorescence, with the effect depending on assay conditions, salt concentration, and the applied β-hairpin peptide. In the absence of agitation, substoichiometric concentrations of the hairpin peptides strongly reduce the lag time of αSyn aggregation, suggesting that they support the nucleation of αSyn amyloid fibrils. The effect is also observed for the aggregation of αSyn fragments lacking the N-terminus or the C-terminus, indicating that the promotion of nucleation involves the interaction of hairpin peptides with the hydrophobic non-amyloid-β component (NAC) region.

## 1. Introduction

The assembly of proteins into amyloid fibrils with cross-β architecture is associated with a wide range of diseases, in particular, neurodegenerative diseases such as Parkinson’s disease (PD) and non-neuropathic systemic amyloidoses such as AL amyloidosis [1]. PD belongs to the synucleinopathies group, which is characterized by aggregates of the protein α-synuclein (αSyn) localized in the brain, whose spreading has been linked to pathogenesis [2,3,4]. In AL amyloidosis, the most prevalent systemic amyloidosis in the Western world, the proliferation of monoclonal plasma cells that secrete high concentrations of immunoglobulin light chains leads to the deposition of light chain amyloid fibrils in various organs, in particular, the heart, kidney, and liver, which is ultimately fatal [5,6].

The nucleation of amyloid fibrils is incompletely understood [7,8]. In the case of αSyn, heterogeneous nucleation processes are thought to dominate, with the surfaces of membranes or of preexisting amyloid fibrils acting as plausible in vivo nucleation sites [9,10,11]. Similar to homologous interactions with αSyn fibrils [10], αSyn monomers can cross-interact with other amyloid proteins such as amyloid-β (Aβ) or islet amyloid polypeptide (IAPP), which may result in the cross-seeding of αSyn aggregation [12,13,14,15].

**Figure 1:**
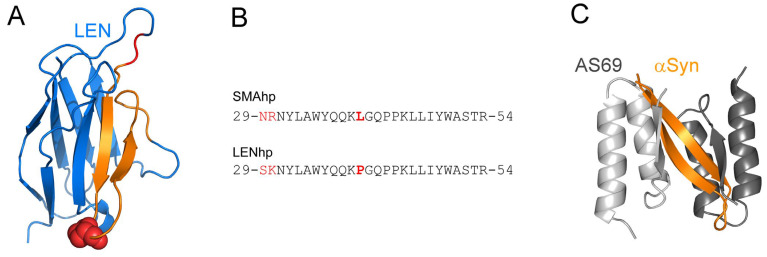
The C-C′ β-hairpin in the light chain variable domain and β-hairpin binding caused by β-wrapins. (**A**) Structure of the LEN VL domain (pdb:1LVE) [54]. The C-C′ β-hairpin is highlighted in orange, apart from the sites that differ between SMA and LEN, which are shown in red. Residue P40 in the turn of the C-C′ β-hairpin is displayed as red spheres. (**B**) Sequences of hairpin peptides investigated in this study, with differences between SMAhp and LENhp highlighted in red. Amino acid residue 40 regulating amyloid formation is printed in bold. (**C**) Structure of β-wrapin AS69 (the two subunits displayed in light gray and dark gray) in complex with the β-hairpin formed by αSyn (orange) (pdb:4BXL) [55]. AS69 binds β-hairpins in the same way as AS10 does [56,57].

The β-hairpin is a structural motif consisting of two β-strands that are connected by a turn and together form an antiparallel β-sheet [16,17]. As β-hairpins and amyloid fibrils are both built from β-strands, β-hairpin conformers are enriched in the conformational ensembles of amyloid-forming proteins [18,19,20,21,22,23,24,25,26,27,28,29,30,31,32,33]. Such β-hairpins may affect amyloid formation, either by forming oligomeric or fibrillar assemblies themselves or by interacting with other oligomers or fibrils [18,19,24,25,27,32,34,35,36,37,38,39,40,41,42,43,44,45,46,47]. β-hairpins may be of particular importance for the amyloid formation of proteins with Greek key folds, including light chains, as these contain anti-parallel β-hairpin loops that are susceptible to diffusion, which may lead to the exposure of sites that subsequently trigger amyloid formation [48]. An interesting case is the β-hairpin formed by the C and C’ β-strands of the light chains SMA and LEN [48,49,50,51,52]. C-C′ β-hairpins in immunoglobulins are only present in the variable domains of light chains (VLs). SMA and LEN are κ4 antibodies that differ in only eight amino acid residues [49]. While SMA is associated with light chain amyloidosis, LEN is not involved in amyloidosis but is connected to multiple myeloma. In line with this, LEN requires harsher conditions, including partial chemical denaturation for amyloid formation in vitro [49,50]. Interestingly, the introduction of a single SMA residue to the LEN sequence, namely, the P40L mutation, is enough to regain the amyloidogenicity of SMA [50]. The P40L mutation is located in the turn connecting the C and C′ β-strands (Figure 1A) [48]. Virtually all human light chain sequences contain proline at position 40, and all those known to have a hydrophobic substitution at position 40 are amyloidogenic [53].

Given their roles in regulating amyloid formation, we chose the C-C′ β-hairpins of SMA and LEN as models to investigate the cross-interactions of β-hairpins and αSyn. The β-hairpins were applied as peptides, referred to as SMAhp and LENhp (Figure 1B). We note that this approach does not imply that C-C′ β-hairpins are accessible as isolated structural units within the sequence context of the full VLs or full light chains under physiological conditions. SMAhp and LENhp are applied here as model β-hairpin-forming peptides. As SMAhp and LENhp lack the flanking polypeptide stretches that stabilize the C-C′ β-hairpin in the immunoglobulin fold, we first validated the potential of the peptides to adopt a β-hairpin structure. To this end, we investigated the interactions of SMAhp and LENhp with the β-hairpin-binding protein β-wrapin AS10, which was previously shown to stabilize β-hairpins in the intrinsically disordered amyloid proteins αSyn, Aβ, and IAPP (Figure 1C) [57]. After validating their potential to adopt a β-hairpin structure, we applied SMAhp and LENhp as model peptides to investigate the cross-interactions of heterologous β-hairpins with the fibril formation of αSyn. Our data indicate that β-hairpin peptides support the nucleation of αSyn amyloid fibrils.

## 2. Results

### 2.1. SMAhp and LENhp Bind to the β-Hairpin-Binding Protein β-Wrapin AS10

The peptides SMAhp and LENhp comprise residues 29–54 of their respective VL domains (Figure 1B). To confirm that these VL segments can indeed adopt a β-hairpin structure in peptide format, we tested their binding to the engineered binding protein β-wrapin AS10. AS10 stems from the phage display selection of a β-wrapin library against the target αSyn and stabilizes a β-hairpin conformation of αSyn (Figure 1C) [55,56,57]. AS10 was found to bind each of the three disease-related amyloid proteins αSyn, Aβ, and IAPP with sub-micromolar affinity, stabilizing related β-hairpins [56,57,58]. AS10 may, therefore, serve as a tool for the identification of β-hairpins that can affect amyloid formation.

**Figure 2:**
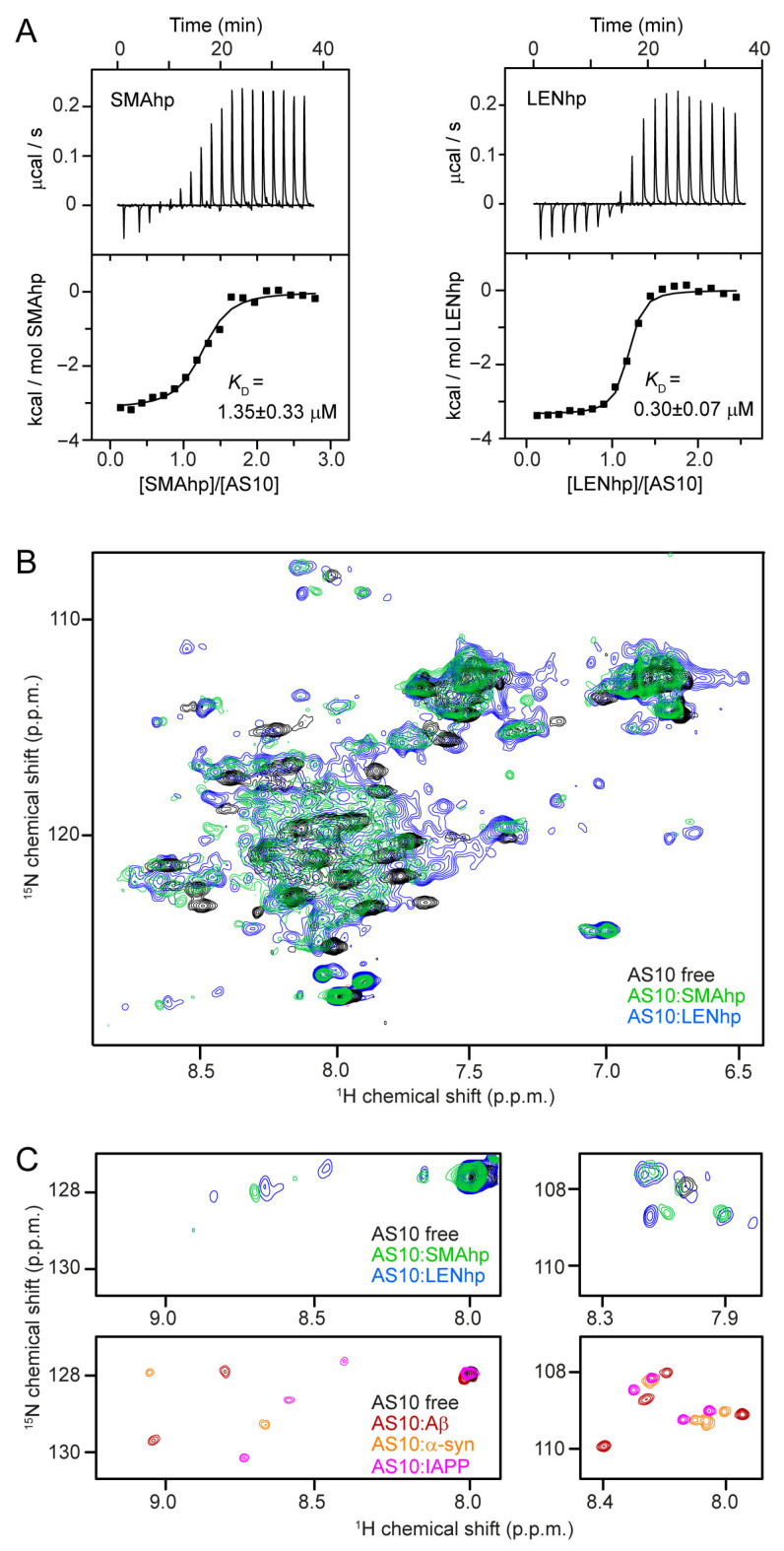
β-hairpin conformation of SMAhp and LENhp upon complex formation with β-wrapin AS10. (**A**) Isothermal titration calorimetry data showing (**top**) the baseline-corrected instrumental response (**bottom**), the integrated data (filled squares), and the best fit of the parameters of a 1:1 binding model (continuous line) upon the titration of 656 µM of SMAhp (**left**) or 600 µM of LENhp (**right**) into 30 µM AS10. (**B**) ^1^H-^15^N HSQC NMR spectrum of ^15^N-AS10 in the absence (black) and presence of a slight molar excess of unlabeled SMAhp (green) or unlabeled LENhp (blue). (**C**) Downfield (**left**) and glycine (**right**) regions of the ^1^H-^15^N HSQC NMR spectra of ^15^N-AS10 in the absence or presence of unlabeled SMAhp (green), LENhp (blue), Aβ (red), αSyn (yellow), or IAPP (magenta).

In isothermal titration calorimetry (ITC), the titration of SMAhp or LENhp into AS10 solutions demonstrated 1:1 binding with dissociation constants of 1.35 ± 0.33 µM or 0.30 ± 0.07 µM, respectively (Figure 2A). These affinities for AS10 are in the same range as those of αSyn (*K*_D_ = 0.38 µM), Aβ (*K*_D_ = 0.15 µM), and IAPP (*K*_D_ = 0.91 µM) [57]. The 4.5-fold higher affinity of LENhp can be explained by the presence of proline residue P40, which is expected to support turn and β-hairpin formation. For both SMAhp and LENhp, endothermic post-transition heat signals were observed, indicating that dilution of the concentrated peptide solutions (c ≈ 0.6 mM) into the ITC cell leads to heat consumption. A plausible explanation is that the peptides form assemblies at high concentrations that (partially) disassemble upon dilution, which would result in the detection of heat from the disassembly reaction.

The structural basis of the interaction of SMAhp and LENhp with AS10 was investigated via ^1^H-^15^N HSQC NMR spectroscopy. Upon the addition of unlabeled SMAhp or LENhp to ^15^N-labeled AS10, the resonance dispersion greatly increased, indicative of coupled folding and binding (Figure 2B). Four amide proton resonances appeared in the glycine region, which originated from Gly-13 and Gly-14 in the two AS10 subunits (Figure 2C). In addition, amide proton resonances were detected in the downfield region of the spectrum with shift values typical of β-sheet conformation (Figure 2C). The same pattern has been observed before for the interaction of AS10 with αSyn, Aβ, and IAPP (Figure 2C) [57]. The NMR data indicate that SMAhp and LENhp adopt a β-hairpin conformation in complex with AS10 analogous to αSyn, Aβ, and IAPP.

### 2.2. SMAhp and LENhp Promote Nucleation of αSyn Amyloid Fibrils

**Figure 3:**
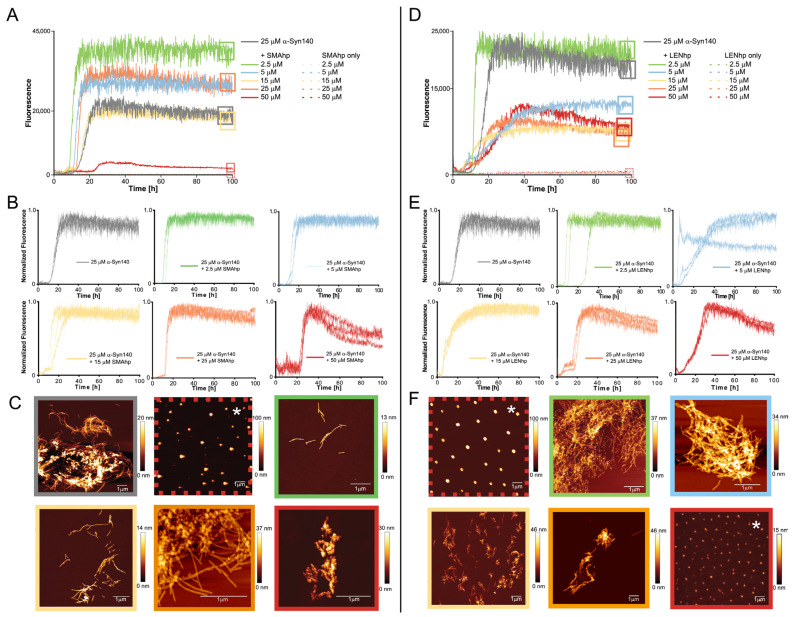
Modulation of αSyn aggregation by SMAhp (**left**) and LENhp (**right**) in ThT assay with agitation. Samples contained one glass bead per microtiter plate well and were continuously shaken at 300 rpm. Buffer, 30 mM Tris-HCl, pH 7.4, 50 mM NaCl. (**A**,**B**,**D**,**E**) Time course of ThT fluorescence of 25 µM αSyn in the absence (gray) and presence of SMAhp (**A**,**B**) or LENhp (**D**,**E**). Panels (**A**,**D**) show one trace per peptide concentration; panels (**B**,**E**) provide triplicate traces to illustrate reproducibility. (**C**,**F**) AFM images of the ThT samples at the end of the aggregation assay. Frames around the images correspond to the frames in panels (**A**,**D**) for indication of the sample; the same color coding for SMAhp and LENhp concentrations as in panels (**A**,**D**) was used for the AFM image frames. AFM images of samples of SMAhp and LENhp in the absence of αSyn are provided in dashed frames. Images that likely display salt crystals as judged by evenly dispersed particles of large height are labeled with an asterisk.

**Figure 4:**
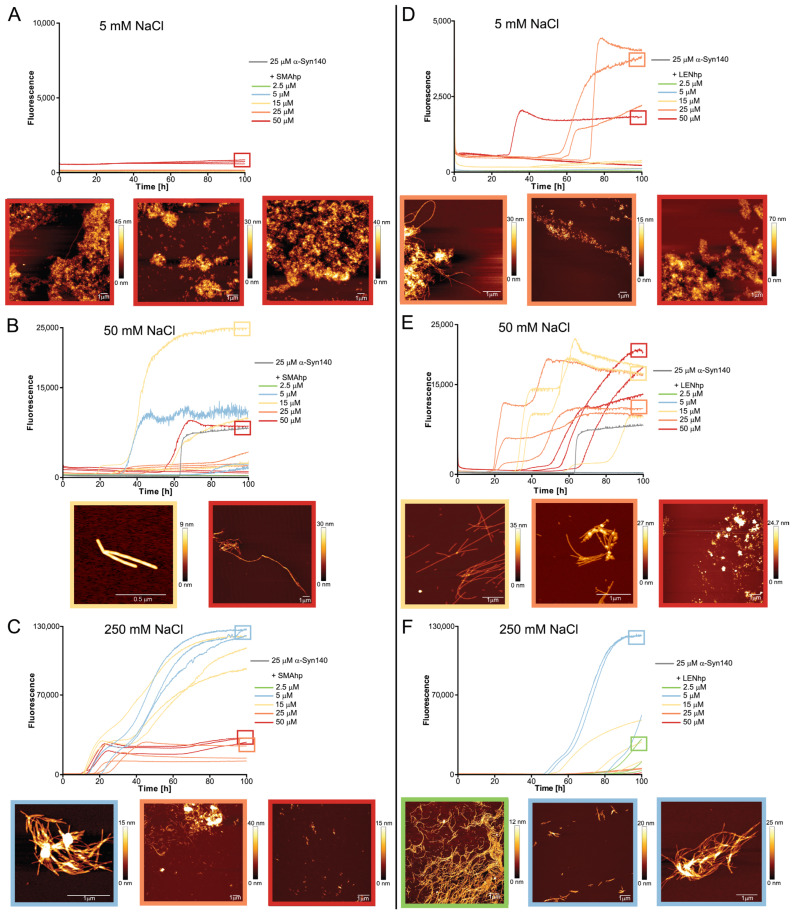
Modulation of αSyn aggregation by SMAhp (**left**) and LENhp (**right**) in ThT assay under quiescent conditions. Buffer, 30 mM Tris-HCl, pH 7.4, with indicated salt concentration. ThT time course and AFM images of 25 µM αSyn in the absence (gray) and presence of SMAhp (**A**–**C**) or LENhp (**D**–**F**). Frames around the AFM images correspond to the frames in the kinetics diagrams for indication of the sample; the same color coding for SMAhp and LENhp concentrations as in the kinetic diagrams was used for the AFM image frames.

**Figure 5:**
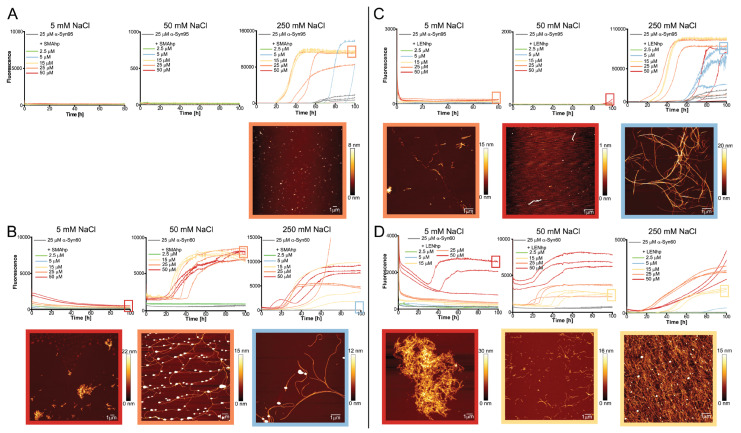
Modulation of aggregation of truncated αSyn variants by SMAhp (**left**) and LENhp (**right**) in ThT assay under quiescent conditions. Buffer, 30 mM Tris-HCl, pH 7.4, with indicated salt concentration. ThT time course and AFM images of (**A**,**C**) 25 µM αSyn(1–95) or (**B**,**D**) 25 µM αSyn(61–140) in the absence (gray) and presence of SMAhp (**A**,**B**) or LENhp (**C**,**D**). Frames around the AFM images correspond to the frames in the kinetics diagrams for indication of the sample; the same color coding for SMAhp and LENhp concentrations as in the kinetic diagrams was used for the AFM image frames.

After validating their potential to adopt a β-hairpin structure, we next tested the effects of SMAhp and LENhp on αSyn fibril formation by recording the kinetics of amyloid formation via the fluorescence of the dye Thioflavin T (ThT) [59]. As the nucleation of αSyn amyloid fibrils is slow at neutral pH, in vitro assays are usually performed under agitation and in the presence of glass beads, which enhances nucleation at the air–water interface and promotes the proliferation of fibrils by increasing the number of fibril ends through fibril fragmentation [11,60,61]. Here, we performed αSyn fibril formation assays both under agitation (Figure 3) and under quiescent conditions (Figure 4 and Figure 5).

In the agitation assay, 25 µM of αSyn exhibited the characteristic sigmoidal time trace of amyloid formation with a lag time of approximately 10 h (Figure 3A,D). In contrast, SMAhp and LENhp alone did not cause an increase in ThT fluorescence (Figure 3A,D). The addition of both SMAhp and LENhp to 25 µM of αSyn had concentration-dependent effects on the ThT time traces. Low concentrations of SMAhp led to a reduction in the lag time, whereas the addition of 50 µM of SMAhp resulted in a prolonged lag time (Figure 3A,B). In contrast, LENhp also reduced the lag time at high concentrations (Figure 3D,E). Biphasic time traces observed for certain concentrations of the hairpin peptides suggest that complex (co-)assembly mechanisms are active. Atomic force microscopy (AFM) showed that αSyn in the absence of hairpin peptides formed long amyloid fibrils with a tendency to cluster (Figure 3C, top row left image). In contrast, the ThT-negative SMAhp and LENhp samples in the absence of αSyn showed particulate structures that were evenly dispersed and of significant height, suggesting that they represent salt crystals (Figure 3C,F, images in red dashed frames; AFM images that likely show salt crystals are labeled with an asterisk). The incubation of αSyn in the presence of SMAhp or LENhp still resulted in amyloid fibrils according to AFM. However, with increasing concentrations of SMAhp and LENhp, the fibril length decreased, and short fibrils clustered into assemblies with an amorphous appearance (Figure 3C,F, bottom rows).

To reduce the impact of fibril fragmentation on the kinetics assays, they were repeated under quiescent conditions (Figure 4). Under these conditions, αSyn fibril formation is usually not observed on the hours-to-days timescale that is commonly covered in in vitro aggregation assays [61]. In line with this, we observed an increase in ThT fluorescence within 100 h only for one of nine samples of αSyn in the absence of hairpin peptides, with high fluorescence occurring after ~60 h in this one case (Figure 4E). The assays were performed for three different salt concentrations ranging from 5 to 250 mM of NaCl in order to identify the potential impact of electrostatic interactions. For all salt concentrations, the addition of β-hairpin peptides resulted in several time courses with increases in ThT fluorescence, with the exception of SMAhp addition to 5 mM of NaCl. The effect was dependent on the peptide concentration, as 2.5 µM of the peptide was usually not sufficient to induce αSyn fibril formation (Figure 4, green time traces). Through all peptide–salt combinations, excluding SMAhp in 5 mM of NaCl, the fraction of aggregation time traces with strong increases in ThT fluorescence at peptide concentrations ≥ 5 µM ranged between 33% (5 mM of NaCl, LENhp, Figure 4D) and 100% (250 mM, SMAhp, Figure 4D). This indicates that hairpin peptides promote αSyn fibril nucleation. The extent of aggregation promotion showed a complex dependency on salt concentration, peptide concentration, and the nature of the peptide, with stronger promotion at 5 and 50 mM of NaCl observed for LENhp and at 250 mM of NaCl for SMAhp.

AFM imaging of the endpoints of the ThT assays frequently detected fibrils but also amorphous material (Figure 4). An increase in the concentrations of the hairpin peptides correlated with reduced fibril length and the appearance of clusters with an amorphous appearance, which were often associated with fibrillar structures (Figure 4; frames around the AFM images are color-coded according to the hairpin peptide concentration from 2.5 to 50 µM in the following order: green, blue, yellow, orange, and red). Amorphous clusters were observed particularly at hairpin peptide concentrations ≥ 25 µM (Figure 4, AFM images in orange or red frames). This concentration dependence of the transition from a fibrillar to apparently amorphous morphology is similar to the one observed in the agitation assay (Figure 3C,F).

### 2.3. Promotion of Nucleation Involves the Hydrophobic Non-Amyloid-β Component (NAC) Segment and the Acidic C-Terminus of αSyn

The dependence of aggregation promotion on salt concentration suggests that electrostatic interactions are involved. At neutral pH, the basic N-terminus of αSyn is positively charged, while the highly acidic C-terminus is negatively charged. SMAhp and LENhp both contain four basic but no acidic residues and could, therefore, engage in electrostatic interactions with the αSyn C-terminus. To evaluate to what extent such interactions account for the observed effects on αSyn aggregation, we investigated the aggregation of truncated αSyn variants lacking the C-terminus, αSyn(1–95), or lacking the N-terminus, αSyn(61–140).

αSyn(1–95) contains the N-terminus and NAC region of αSyn. SMAhp and LENhp reduced the lag time of the fibril formation of αSyn(1–95) in the presence of 250 mM of NaCl, an effect that was strongly dependent on the hairpin peptide concentration (Figure 5A,C). At low salt concentrations, only LENhp addition led to a late increase in ThT fluorescence, accompanied by fibril formation according to AFM (Figure 5C). This indicates that the aggregation-promoting effects of the hairpin peptides are indeed reduced in the absence of the αSyn C-terminus.

αSyn(61–140) contains the NAC region and C-terminus of αSyn. SMAhp and LENhp strongly promote the aggregation of αSyn(61–140) based on ThT fluorescence (Figure 5B,D). Interestingly, at low salt concentrations, ThT fluorescence intensity was already high at the start of the aggregation assay and increased with the concentration of the hairpin peptide. This indicates that, under these conditions, which favor electrostatic interactions, the β-hairpin peptides instantly form ThT-positive assemblies together with αSyn(61–140). Subsequent increases in ThT fluorescence later in the aggregation assay suggest that alternative fibril assemblies with increased thermodynamic stability were formed. As a further test of the importance of electrostatic interactions, we added the polyamine spermine to αSyn(61–140). Spermine has a net charge of +4 at neutral pH, just like SMAhp and LENhp, and has been shown to promote αSyn fibril formation in assays employing agitation conditions [62]. Under the quiescent conditions applied here, spermine at concentrations up to 50 µM (i.e., in the same concentration range covered for SMAhp and LENhp) did not lead to any increase in ThT fluorescence. This suggests that it is not just the presence of charges but also the interplay between polypeptide chains that governs the consequences of this cross-interaction for αSyn assembly.

The data obtained for the truncated αSyn variants suggest that both the hydrophobic NAC region and the acidic C-terminus of αSyn are involved in the promotion of αSyn nucleation by SMAhp and LENhp.

### 2.4. SMAhp and LENhp Modulate the Kinetics of Aβ Aggregation

**Figure 6:**
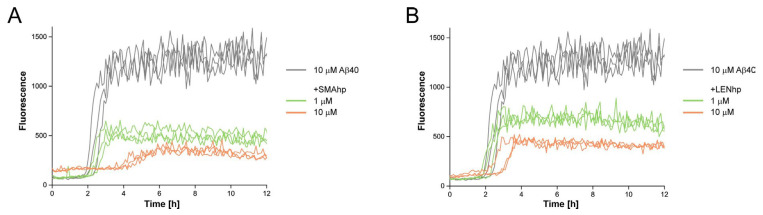
Modulation of Aβ40 aggregation by SMAhp (**left**) and LENhp (**right**) in ThT assay with agitation. Samples contained one glass bead per microtiter plate well and were continuously shaken at 300 rpm. Buffer, 20 mM Na-phosphate, pH 7.4, 50 mM NaCl. Triplicates of time courses of ThT fluorescence of 10 µM Aβ40 in the absence (gray) and presence of SMAhp (**A**) or LENhp (**B**).

To test if the modulating effects of SMAhp and LENhp on protein aggregation were specific to αSyn, we finally studied the kinetics of Aβ40 aggregation in an agitation assay. The addition of stoichiometric amounts of SMAhp or LENhp to 10 µM of Aβ40 resulted in a prolonged lag time (Figure 6A,B). In contrast to αSyn, a reduction in the lag time at low concentrations of SMAhp or LENhp was not discernable. This suggests that these β-hairpin peptides have a general capacity to cross-interact with heterologous amyloid-forming proteins. However, the current data do not reveal if the same mechanistic basis applies to the modulation of αSyn and Aβ40 aggregation.

## 3. Discussion

In this work, we chose to investigate the C-C′ β-hairpins of the SMA and LEN light chains as models to test for the potential cross-interaction of β-hairpins with αSyn fibril formation. The choice was based on the observation that hairpin formation in SMA and LEN might be critical for their involvement in AL amyloidosis vs. multiple myeloma, as the identity of the turn residue at position 40 regulates amyloidogenicity [50]. Interestingly, LENhp, which contains the turn-promoting proline at position 40, binds to the β-hairpin-binding protein AS10 with a similar sub-micromolar affinity to αSyn, Aβ, and IAPP. The affinity of SMAhp is weaker, with a 4.5-fold higher *K*_D_. Importantly, affinity to AS10 is not a sequence-unspecific property of any disordered polypeptide. For example, neither the four-repeat-domain tau protein construct K18ΔK280/AA nor the Y145Stop variant of human prion protein huPrP (23–144) exhibit affinity for AS10 [57]. Thus, with regard to AS10 binding, SMAhp and LENhp have similar properties to the β-hairpin regions involved in the oligomer and fibril formation of αSyn, Aβ, and IAPP.

Different activities of β-hairpins with respect to amyloid formation have been described based on simulations and experiments. β-hairpins may act as on-pathway intermediates, possibly even as primary nuclei [19,32,35]. On the other hand, they may also prevent fibril formation [34,36,37,39,40,42]. With regard to cross-interactions, experimental evidence was obtained regarding the key role of β-hairpins in the cross-seeding of IAPP fibril formation caused by a prion protein fragment and by a Tau fragment [43,47]. In line with this, we find here that the light chain β-hairpin peptides SMAhp and LENhp promote αSyn fibril nucleation, resulting in αSyn fibril formation under quiescent conditions at neutral pH, where this is usually not observed. Our data indicate that both the hydrophobic NAC region and the acidic C-terminus are responsible for this activity. However, a detailed understanding of the mechanism was not achieved based on the present data. SMAhp and LENhp might act as monomers since they are available as monomers, as demonstrated by the formation of their 1:1 complexes with AS10 in ITC. Such activity on the level of monomeric peptides would not be considered cross-seeding, as it would not depend on the presence of a templating–competent protein aggregate. On the other hand, the post-transition heats of dilution in ITC suggest that the peptides can form oligomers. Therefore, it may also be the oligomeric assemblies of the β-hairpin peptides that provide the interaction surface for the heterogeneous nucleation of αSyn fibrils, i.e., for cross-seeding. AFM imaging showed that SMAhp and LENhp alter the morphology of αSyn aggregates, with a shift from the formation of long fibrils to clusters of short fibrils and amorphous material at (close to) stoichiometric concentrations of β-hairpin peptides. While this is further evidence of the profound modulation of αSyn aggregation reactions, the molecular basis of the shift in aggregate morphology requires further investigation.

SMAhp and LENhp show overall similar activities in promoting αSyn fibril formation under quiescent conditions at neutral pH, although some differences exist; SMAhp is a stronger promoter at high salt concentration but a weaker promoter at low salt concentration (Figure 4). The reason for this difference was not revealed by the present data, and it is unclear if a difference in β-hairpin propensity due to the different amino acids at position 40 is involved.

An interesting unknown is the extent to which the cross-interactions of β-hairpins are specific to certain protein:β-hairpin pairs. We find that SMAhp and LENhp modulate both αSyn and Aβ40 aggregation, suggesting that certain β-hairpin peptides may have a general capacity to interfere with amyloid formation. However, to elucidate this further, a better mechanistic understanding is required, and individual protein:β-hairpin pairs should be investigated in detail.

In conclusion, we find that model β-hairpin peptides derived from disease-related immunoglobulin light chains can cross-interact with αSyn to promote αSyn fibril nucleation.

## 4. Materials and Methods

### 4.1. Peptides

SMAhp and LENhp were obtained as synthetic peptides from CASLO. Aβ40 was obtained from Bachem. To fully monomerize the peptides, 1 mg aliquots were solubilized in 1 mL hexafluoroisopropanol (HFIP), aliquoted in smaller amounts, lyophilized, and stored at −20 °C.

### 4.2. Proteins

Full-length αSyn was expressed and purified as described previously, yielding N-terminally acetylated protein [59,63].

αSyn(1–95) and αSyn(61–140) were expressed from codon-optimized gene sequences in the pT7–7 vector in *Escherichia coli* BL21DE3, without N-terminal acetylation. Expression and purification were performed as for full-length αSyn, with the difference that, for αSyn(1–95), the anion exchange chromatography step was replaced by cation exchange chromatography using a 5 mL HiTrap SP FF column (Cytiva, Marlborough, MA, USA) and 10 mM sodium acetate buffer, pH 5, with elution in a NaCl gradient from 0 to 500 mM.

β-wrapin AS10 with or without isotope labels for NMR spectroscopy was expressed and purified as previously described [55,57].

### 4.3. Isothermal Titration Calorimetry

ITC was performed on a Microcal iTC200 calorimeter (GE Healthcare, Chicago, IL, USA). A temperature of 30 °C was chosen, which was previously found to yield high-quality ITC data for the AS10:αSyn interaction [57]. The cell was filled with AS10 at a concentration of 30 μM in 20 mM sodium phosphate, pH 7.4, 50 mM NaCl. The syringe was filled with 656 μM SMAhp or 600 μM LENhp followed by titration of the β-hairpin peptide solutions into the cell. Heats of post-saturation injections were averaged and subtracted from each injection to correct for heats of dilution and mixing. Dissociation constants were obtained from a nonlinear least-squares fit to a 1:1 binding model using MicroCal Origin.

### 4.4. NMR Spectroscopy

NMR spectroscopy was performed on a VNMRS instrument (Varian, Las Vegas, NV, USA) at a proton frequency of 800 MHz, equipped with a cryogenically cooled Z-axis pulsed field gradient (PFG) triple resonance probe. A temperature of 25 °C was chosen to allow for comparison with previously recorded data for the AS10 interactions with αSyn, Aβ, and IAPP [57]. SMAhp and LENhp were not isotopically labeled and added in a slight excess to ^15^N-labeled AS10. The sample buffer was 20 mM sodium phosphate, pH 7.4, 50 mM NaCl, supplemented with 10% D2O. NMR data were processed using NMRPipe [64] and analyzed with CcpNmr [65].

### 4.5. Thioflavin T Aggregation Assay

Aggregation kinetics were monitored in Greiner 96-well half-area, clear bottom, low-binding plates via ThT fluorescence in a BMG CLARIOstar plate reader. A temperature of 37 °C was chosen for physiological relevance. Previous studies have shown that ITC data from β-wrapins obtained at 30 °C correlate well with effects on aggregation at 37 °C [66]. Wells with samples were always surrounded by wells filled with liquid, either other samples or 150 µL water, to counter potential artifacts due to evaporation. The assays conducted under agitation were performed with continuous orbital shaking at 300 rpm and with the addition of one glass bead (0.75–1 mm, Roth) per sample well. αSyn, αSyn(1–95), and αSyn(61–140) were applied at a constant concentration of 25 µM in 30 mM Tris-HCl, pH 7.4, 25 µM ThT, 0.04% NaN_3_ and 5, 50, or 250 mM NaCl. SMAhp and LENhp were dissolved at a high concentration in 30 mM Tris-HCl, pH 7.4, 50 mM NaCl and added to the sample wells to achieve final concentrations between 2.5 and 50 µM. Samples were prepared in triplicates. Plates were covered with sealing tape, placed in the plate reader, heated to 37 °C, and ThT fluorescence was read using bottom optics.

### 4.6. Atomic Force Microscopy

For AFM imaging, the samples were taken out of the 96-well plates after the ThT aggregation assay. In total, 5 μL of each sample was placed on a freshly cleaved muscovite mica surface and incubated for 2 min. Subsequently, the samples were washed with 100 μL ddH2O three times and dried with a stream of N_2_ gas. Imaging was performed in intermittent contact mode (AC mode) with a JPK NanoWizard 3 atomic force microscope (JPK) using a silicon cantilever with a silicon tip (OMCL-AC160TS-R3, Olympus, Tokyo, Japan), with a typical tip radius of 9 ± 2 nm, a force constant of 26 N/m, and a resonance frequency of around 300 kHz. The images were processed using the JPK Data Processing Software (version spm-5.0.84).

## Data Availability

The data are contained within the article.
